# Microstructure-Driven Loss Mechanisms and Tensor-Based FEM Calibration

**DOI:** 10.3390/mi17070850

**Published:** 2026-07-17

**Authors:** Annamaria Muoio, Angela Garofalo, Francesco La Via

**Affiliations:** 1 Institute for Microelectronics and Microsystems(IMM), National Research Council (CNR), Strada VIII, 5, 95121 Catania, Italy; 2 Materials Science Department, Milano—Bicocca University, Via R. Cozzi 55, 20125 Milan, Italy; a.garofalo22@campus.unimib.it

**Keywords:** 3C-SiC, SiC microsystem, heteroepitaxial SiC, residual stress, microresonators, anisotropic damping, quality factor (Q-factor), finite element method (FEM), resonators, strain sensors, harsh environment

## Abstract

Silicon carbide (SiC) is a key material for next-generation miniaturized devices and MEMS operating in harsh environments. This paper presents a comprehensive investigation of anisotropic damping mechanisms in heteroepitaxial 3C-SiC double-clamped beam resonators for MEMS applications. Unlike conventional isotropic loss-factor models, which assign a single scalar damping coefficient to all deformation directions, the proposed framework employs a full 6 × 6 loss-factor tensor expressed in Voigt notation, implemented within the COMSOL Multiphysics finite element environment. The tensor formulation enables the direction-dependent description of energy dissipation, capturing the coupling between shear and normal strain modes that arises from the (111) crystallographic orientation and from the heteroepitaxial defect structure of 3C-SiC grown on silicon substrates. The effects of film thickness, effective Young’s modulus, and residual stress on elastic modulus, resonance frequency, and Q-factor are systematically analyzed across five wafers (w1–w5, thickness range 293–890 nm). Experimentally calibrated anisotropic loss-factor matrices are extracted via least-squares fitting to measured Q-factors, and their Frobenius norms are found to correlate negatively with resonance frequency. The anisotropic model reduces Q-factor prediction errors to below 1% for all wafers, significantly outperforming the isotropic approach, particularly for films thicker than 600 nm. These results demonstrate that an accurate treatment of directional dissipation is essential for the design of high-Q resonators and high-sensitivity strain sensors targeted at geophysical monitoring applications.

## 1. Introduction

Silicon carbide (SiC) is recognized as a material with exceptional potential for the design of microelectromechanical systems (MEMSs), especially in harsh environments and applications requiring long-term stability. Among the various SiC polytypes, cubic 3C-SiC stands out due to its compatibility with silicon substrates, excellent mechanical robustness, and scalable manufacturing on large areas at reduced cost. These characteristics make 3C-SiC highly suitable for advanced MEMS technologies, such as seismic and volcanic monitoring systems, high-temperature sensors, and high-sensitivity strain gauges [1]. Despite its many advantages, the full potential of 3C-SiC in MEMS is significantly limited by the structural quality of heteroepitaxial films grown on silicon. The large lattice and thermal mismatch between 3C-SiC and Si inevitably leads to high defect densities, residual stresses, and non-uniform stress gradients within the film. These material features directly affect the mechanical behavior of MEMS devices, causing variations in critical parameters such as Young’s modulus, resonance frequency, energy dissipation mechanisms, and ultimately the Q-factor. A central challenge in modeling these devices is the accurate representation of mechanical damping. Understanding how these properties evolve as a function of thickness, doping, crystallographic orientation and growth conditions is therefore essential [2,3,4]. While a single scalar loss factor may approximate average dissipation, it cannot represent direction-dependent mechanisms arising from crystal symmetry, defect distribution, and stress anisotropy. In this context, anisotropic models based on Voigt notation and 6 × 6 loss-factor matrices provide a far more accurate representation of energy dissipation, allowing mode-dependent Q-factors and frequency trends to be predicted more reliably. The present work builds on the framework introduced in a previous conference contribution [5], extending it with a rigorous mathematical derivation, a detailed physical interpretation of the fitted damping tensors, uncertainty quantification, and strain sensitivity analysis. Finite element analysis (FEA), calibrated on experimentally derived anisotropic matrices, has shown the significant influence of shear-mode dissipation, defect-induced anisotropy, and residual stress on the dynamic response of 3C-SiC MEMS resonators. Integrating experimental characterization with numerical modeling proves essential for evaluating the mechanical behavior of 3C-SiC films and guiding MEMS design. By correlating film quality, stress state, defect evolution, and damping properties, more accurate predictive models can be developed, enabling the design of high-Q resonators and highly sensitive strain sensors [6]. This work provides a comprehensive overview of heteroepitaxial 3C-SiC for MEMS applications, discussing growth mechanisms, defect evolution, mechanical properties, stress behavior, and recent modeling approaches for energy dissipation, with particular emphasis on anisotropic damping behavior. This paper is organized as follows: Section 2 introduces the theoretical framework, Section 3 describes the FEM model and calibration procedure, Section 4 presents the results and discussion, and Section 5 summarizes the main conclusions.

## 2. Theoretical Framework: Viscoelastic Damping and Voigt Notation

Energy dissipation in such devices is commonly described by phenomenological viscoelastic models, in which the elastic stiffness is supplemented by a loss factor that accounts for internal friction and other microscopic dissipation mechanisms. In contrast, the crystalline symmetry of 3C-SiC and the residual stress state of epitaxial films naturally lead to an anisotropic loss mechanism. In this case the loss factor is more appropriately described by a full 6×6 tensor in Voigt notation, which couples the different stress and strain components. Understanding and quantifying this anisotropic damping is essential for explaining mode-dependent Q-factors and for guiding the design of future high-Q MEMS resonators. Table 1 summarizes the key differences between the two modeling strategies.

### 2.1. Physical Relevance of Stiffness-Tensor Rotation for (111) 3C-SiC

The rotation of the stiffness tensor is not just a formal mathematical operation but a physically essential step for correctly modeling (111)-oriented 3C–SiC. This reflects the fact that the (111) plane is oblique with respect to the principal cubic axes, so that shear deformation along $\langle 1\bar{1}0\rangle$ and $\langle 11\bar{2}\rangle$ directions couples directly to normal strain components. If this rotation were omitted, the FEM model would incorrectly assume that shear and normal strains evolve independently, which is inconsistent with the deformation mechanisms observed in heteroepitaxial 3C–SiC films under flexural loading. To make this explicit, the rotated tensor stiffness matrix $C^{\left(111\right)}$ in Voigt notation acquires additional non-zero off-diagonal terms. A representative form is

(1) $$C^{\left(111\right)}=\left(\begin{array}{cccccc} C_{11}^{\prime } & C_{12}^{\prime } & C_{13}^{\prime } & C_{14}^{\prime } & C_{15}^{\prime } & C_{16}^{\prime }\\ C_{12}^{\prime } & C_{11}^{\prime } & C_{13}^{\prime } & -C_{14}^{\prime } & C_{16}^{\prime } & -C_{15}^{\prime }\\ C_{13}^{\prime } & C_{13}^{\prime } & C_{33}^{\prime } & 0 & 0 & 0\\ C_{14}^{\prime } & -C_{14}^{\prime } & 0 & C_{44}^{\prime } & C_{45}^{\prime } & C_{46}^{\prime }\\ C_{15}^{\prime } & C_{16}^{\prime } & 0 & C_{45}^{\prime } & C_{44}^{\prime } & C_{56}^{\prime }\\ C_{16}^{\prime } & -C_{15}^{\prime } & 0 & C_{46}^{\prime } & C_{56}^{\prime } & C_{44}^{\prime } \end{array}\right)$$

The appearance of terms such as the components $C_{14}^{\prime }$, $C_{15}^{\prime }$ and $C_{16}^{\prime }$ of the rotated stiffness tensor $C^{\left(111\right)}$ directly reflects the orientation-induced coupling between shear and normal deformation. As shown in Equation (1), the rotated stiffness tensor $C^{\left(111\right)}$ contains non-zero shear-normal coupling terms that originate from the (111) crystallographic orientation.

### 2.2. Modeling Anisotropic Damping in Viscoelastic Materials Using the Voigt Framework

The Voigt model describes the viscoelastic behavior of materials using an elastic spring and a viscous damper arranged in parallel. For an uniaxial stress state, the constitutive relation in the time domain is

(2) $$\sigma \left(t\right)=E\;\varepsilon \left(t\right)+\eta \;\dot{\varepsilon }\left(t\right),$$

where σ(t) is the stress, ε(t) is the strain, *E* is the elastic modulus, and η is the viscosity coefficient [7].

For harmonic loading at angular frequency ω, the stress and strain can be written as

(3) $$\varepsilon \left(t\right)=ℜ\left\{\hat{\varepsilon }e^{i\omega t}\right\},\;\sigma \left(t\right)=ℜ\left\{\hat{\sigma }e^{i\omega t}\right\},$$

which leads to a complex modulus

(4) $$\hat{\sigma }=E^{*}\hat{\varepsilon },\;E^{*}=E^{\prime }+iE^{\prime \prime }.$$

The loss factor is defined as

(5) $$\eta =\frac{E^{\prime \prime }}{E^{\prime }}.$$

In a hysteretic damping model, η is taken as frequency-independent, and the complex modulus is written as

(6) $$E^{*}=E\left(1+i\eta \right),$$

where *E* is the storage modulus and η quantifies the energy dissipated per cycle [8]. A frequency-independent hysteretic model [9,10], is adopted because the dissipation mechanisms that dominate (111) 3C-SiC resonators in the 200–350 kHz range are governed by extended defects (stacking faults, partial dislocations, micro-twins) whose relaxation times are much shorter than the oscillation period. In this limit, the material exhibits structural damping, characterized by a constant loss factor independent of frequency. This behavior is widely observed in crystalline MEMS resonators operating below the MHz regime and has been reported for SiC, Si, GaN, and other high-Q single-crystal materials. The Q-values measured experimentally across all thicknesses show negligible frequency dependence, confirming no transition between distinct relaxation regimes occurs in the studied range. The hysteretic model implemented in COMSOL is therefore consistent with both established viscoelastic theory and the experimental evidence collected for 3C-SiC films of thickness between 293 and 890 nm.

### 2.3. Tensor and Voigt Notation

In this work, we adopt the conventional Voigt mapping

(7) $$\sigma ={\left[\begin{array}{cccccc} \sigma_{11} & \sigma_{22} & \sigma_{33} & \sigma_{23} & \sigma_{13} & \sigma_{12} \end{array}\right]}^{T},\;\varepsilon ={\left[\begin{array}{cccccc} \varepsilon_{11} & \varepsilon_{22} & \varepsilon_{33} & \gamma_{23} & \gamma_{13} & \gamma_{12} \end{array}\right]}^{T},$$

where the engineering shear strains are defined as $\gamma_{ij}=2\varepsilon_{ij}$. With this convention the 6×6 stiffness matrix *C* collects all independent components of the fourth-order tensor $C_{ijkl}$ and the shear terms in *C* already include the factor of 2 relating $\gamma_{ij}$ to $\varepsilon_{ij}$. This choice is consistent with the implementation used in COMSOL and avoids ambiguity in the definition of the loss-factor matrix η introduced below. For a general 3D anisotropic material, the linear elastic constitutive relation is

(8) $$\sigma_{ij}=C_{ijkl}\;\varepsilon_{kl},$$

where $C_{ijkl}$ is the fourth-order stiffness tensor. Using Voigt notation, the pair of indices ij and kl is mapped to single indices I,J=1,…,6, and the relation becomes

(9) σ=Cε,

with C a 6×6 stiffness matrix, $\sigma ={\left(\sigma_{11},\sigma_{22},\sigma_{33},\sigma_{23},\sigma_{13},\sigma_{12}\right)}^{T}$ and $\varepsilon ={\left(\varepsilon_{11},\varepsilon_{22},\varepsilon_{33},\gamma_{23},\gamma_{13},\gamma_{12}\right)}^{T}$. The symmetry of the stress tensor $\sigma_{ij}$ means that there are at most 6 different elements of stress. Similarly, there are at most six different elements of the strain tensor $\varepsilon_{ij}$. The mapping is as follows:

(10) $$\left[\begin{array}{c} \sigma_{1}=\sigma_{11}\\ \sigma_{2}=\sigma_{22}\\ \sigma_{3}=\sigma_{33}\\ \sigma_{4}=\sigma_{23}\\ \sigma_{5}=\sigma_{13}\\ \sigma_{6}=\sigma_{12} \end{array}\right]and\left[\begin{array}{c} \varepsilon_{1}=\varepsilon_{11}\\ \varepsilon_{2}=\varepsilon_{22}\\ \varepsilon_{3}=\varepsilon_{33}\\ \varepsilon_{4}=\varepsilon_{23}\\ \varepsilon_{5}=\varepsilon_{13}\\ \varepsilon_{6}=\varepsilon_{12} \end{array}\right]=>\left[\begin{array}{c} \sigma_{11}\\ \sigma_{22}\\ \sigma_{33}\\ \sigma_{23}\\ \sigma_{13}\\ \sigma_{12} \end{array}\right]=\left[\begin{array}{cccccc} C_{11} & C_{12} & C_{13} & C_{14} & C_{15} & C_{16}\\ C_{12} & C_{22} & C_{23} & C_{24} & C_{25} & C_{26}\\ C_{13} & C_{23} & C_{33} & C_{34} & C_{35} & C_{36}\\ C_{14} & C_{24} & C_{34} & C_{44} & C_{45} & C_{46}\\ C_{15} & C_{25} & C_{35} & C_{45} & C_{55} & C_{56}\\ C_{16} & C_{26} & C_{36} & C_{46} & C_{56} & C_{66} \end{array}\right]\left[\begin{array}{c} \varepsilon_{11}\\ \varepsilon_{22}\\ \varepsilon_{33}\\ 2\varepsilon_{23}\\ 2\varepsilon_{31}\\ 2\varepsilon_{12} \end{array}\right]$$

The Voigt mapping reported in Equation (10) is adopted throughout this work for both the elastic stiffness tensor and the anisotropic loss-factor matrix. In accordance with the conventional Voigt notation used in COMSOL, the shear strain components are represented as engineering strains ($\gamma_{ij}=2\varepsilon_{ij}$). Therefore, the last three terms of the strain vector include the factor of 2 associated with shear deformation. From symmetry considerations, the number of independent moduli can be determined for various crystal classes. A cubic crystal has only three independent elastic constants; these may be written compactly in the following matrix, giving the stress–strain relations

(11) $$\left[\begin{array}{c} \sigma_{11}\\ \sigma_{22}\\ \sigma_{33}\\ \sigma_{23}\\ \sigma_{13}\\ \sigma_{12} \end{array}\right]=\left[\begin{array}{cccccc} C_{11} & C_{12} & C_{12} & 0 & 0 & 0\\ C_{12} & C_{11} & C_{12} & 0 & 0 & 0\\ C_{12} & C_{12} & C_{11} & 0 & 0 & 0\\ 0 & 0 & 0 & C_{44} & 0 & 0\\ 0 & 0 & 0 & 0 & C_{44} & 0\\ 0 & 0 & 0 & 0 & 0 & C_{44} \end{array}\right]\left[\begin{array}{c} \varepsilon_{11}\\ \varepsilon_{22}\\ \varepsilon_{33}\\ 2\varepsilon_{23}\\ 2\varepsilon_{31}\\ 2\varepsilon_{12} \end{array}\right]$$

There is no coupling between shear and tensile components; a tensile strain cannot produce a shear stress and vice versa. This behavior is evident from the cubic stiffness matrix reported in Equation (11), where all shear-normal coupling terms vanish. Cubic crystals such as 3C-SiC exhibit a high degree of symmetry: the stiffness matrix has only three independent elastic constants ($C_{11},C_{12},C_{44}$). For practical FEM implementation in the frequency domain, the constitutive law is expressed as

(12) $$\sigma =C^{*}:\varepsilon ,\;C^{*}=C+i\left(C^{o}H\right)$$

where H is the hysteretic loss factor:

– Isotropic case: $H=\eta I_{6x6}$ (single scalar applied uniformly),
– Anisotropic case: $H={\left[\eta_{IJ}\right]}_{6x6}$ (Voigt notation), allowing directional damping.

Cubic crystals such as 3C-SiC exhibit a high degree of symmetry: the stiffness matrix has only three independent elastic constants and can be written in compact form. In the present work, we use literature values for the elastic constants of 3C-SiC and transform them to the appropriate crystal orientation for (111)-oriented films [8,11]. The loss-factor matrix $\eta_{IJ}$ introduced in the viscoelastic formulation is not arbitrary: it is constrained by the thermodynamic requirements of linear irreversible processes and by the crystallographic symmetry of 3C-SiC. In non-magnetic materials and in the absence of external fields, the dissipative part of the constitutive law must satisfy the Onsager reciprocal relations, which impose $\eta_{IJ}=\eta_{JI}$. This ensures that the internal dissipation per cycle remains positive, in agreement with the Clausius–Duhem inequality and Onsager reciprocal relations [12,13]. Equation (12) provides the general frequency-domain constitutive framework that is later specialized to isotropic and anisotropic damping formulations.

### 2.4. Isotropic Versus Anisotropic Loss Factors

The rotation of the stiffness tensor from the cubic native frame to the (111) wafer coordinate system is not merely a formal operation—it is a physically essential step that directly affects the predictive accuracy of the FEM model. Although cubic 3C–SiC has only three independent elastic constants in its native frame, the transformation to the (111) orientation mixes shear and normal components, producing an anisotropic effective stiffness even for a nominally cubic material. The (111) plane is oblique with respect to the principal cubic axes so that shear along $\langle 1\bar{1}0\rangle$ and $\langle 11\bar{2}\rangle$ couples directly to normal strain components. If this rotation were omitted, the FEM model would incorrectly assume that shear and normal strains evolve independently, contradicting the deformation mechanisms observed in heteroepitaxial 3C–SiC films under flexural loading.

(13) $$C^{\prime }={\mathrm{TCT}}^{T},$$

The transformation matrix T maps the cubic basis ([100],[010],[001]) onto the wafer axes $\left(\left[1\bar{1}0\right],\left[11\bar{2}\right],\left[111\right]\right)$. The three wafer directions are first normalized:

(14) $$e_{1}=\frac{1}{\sqrt{2}}\left(1,-1,0\right),\;e_{2}=\frac{1}{\sqrt{6}}\left(1,1,-2\right),\;e_{3}=\frac{1}{\sqrt{3}}\left(1,1,1\right).$$

These unit vectors form the rows of the rotation matrix:

(15) $$T=\left(\begin{array}{ccc} \frac{1}{\sqrt{2}} & -\frac{1}{\sqrt{2}} & 0\\ \frac{1}{\sqrt{6}} & \frac{1}{\sqrt{6}} & -\frac{2}{\sqrt{6}}\\ \frac{1}{\sqrt{3}} & \frac{1}{\sqrt{3}} & \frac{1}{\sqrt{3}} \end{array}\right)$$

The stiffness tensor is rotated through the standard fourth-order transformation:

(16) $$C_{ijkl}^{\prime }=a_{ip}\;a_{jq}\;a_{kr}\;a_{ls}\;C_{pqrs}$$

where $a_{ip}$ are the entries of T. This procedure produces the non-zero off-diagonal components $C_{14}^{\prime },\;C_{15}^{\prime },\;C_{16}^{\prime }$ observed in the (111) stiffness matrix, which originate from the crystallographic misalignment between the wafer axes and the cubic frame. Providing T explicitly makes the rotation fully reproducible and enables direct verification of all transformed stiffness coefficients. This rotation is physically essential: although 3C-SiC is nominally cubic, the (111) cut induces coupling between normal and shear strains because the (111) plane is oblique to the principal cubic directions in the presence of heteroepitaxial defects such as stacking faults and partial dislocations—which predominantly lie along {111} planes. If the rotation were omitted, the FEM model would artificially enforce a separation between shear and normal deformation modes, contradicting the actual mechanical behavior of (111)-oriented 3C-SiC films under flexural excitation. The effect of the rotation can be illustrated by the structure of the transformed stiffness tensor. In Voigt notation, the rotated tensor $C^{\left(111\right)}$ acquires additional non-zero off-diagonal terms:

(17) $$C^{\left(111\right)}=\left(\begin{array}{cccccc} C_{11}^{\prime } & C_{12}^{\prime } & C_{13}^{\prime } & C_{14}^{\prime } & C_{15}^{\prime } & C_{16}^{\prime }\\ C_{12}^{\prime } & C_{11}^{\prime } & C_{13}^{\prime } & -C_{14}^{\prime } & C_{16}^{\prime } & -C_{15}^{\prime }\\ C_{13}^{\prime } & C_{13}^{\prime } & C_{33}^{\prime } & 0 & 0 & 0\\ C_{14}^{\prime } & -C_{14}^{\prime } & 0 & C_{44}^{\prime } & C_{45}^{\prime } & C_{46}^{\prime }\\ C_{15}^{\prime } & C_{16}^{\prime } & 0 & C_{45}^{\prime } & C_{44}^{\prime } & C_{56}^{\prime }\\ C_{16}^{\prime } & -C_{15}^{\prime } & 0 & C_{46}^{\prime } & C_{56}^{\prime } & C_{44}^{\prime } \end{array}\right)$$

**Figure 1:**
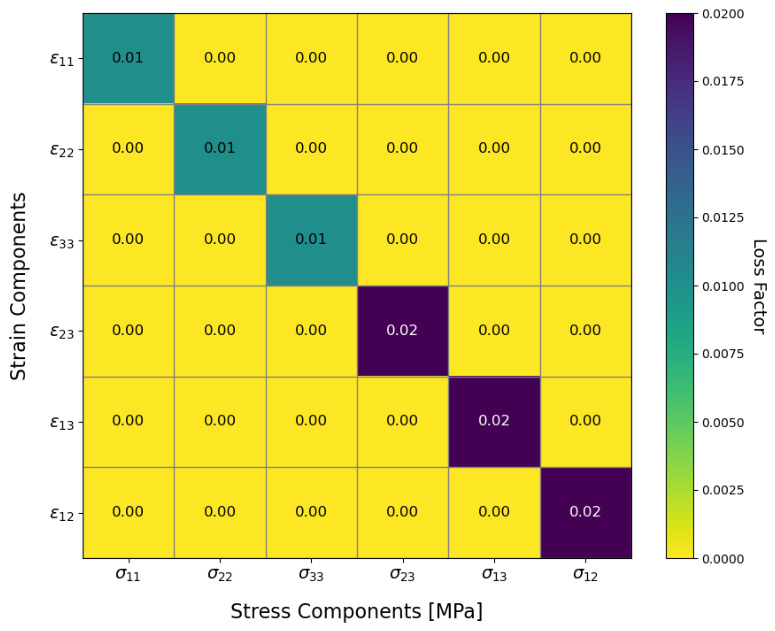
Anisotropic loss factor matrix expressed in Voigt notation, with strain components ($\varepsilon_{ij}$) on the vertical axis and stress components ($\sigma_{ij}$) on the horizontal axis.

The structure of Equation (17) highlights the appearance of orientation-induced coupling terms that are absent in the native cubic coordinate system. These terms represent the intrinsic mixing between shear and normal deformation modes and constitute the structural foundation upon which the anisotropic loss-factor formulation is built. For this reason, the rotation of the stiffness tensor must be performed prior to defining the complex stiffness tensor $C=C^{\prime }\left(I+i\eta \right)$. Before comparing isotropic and anisotropic formulations, we recall that the loss-factor matrix η introduced in Equation (20) is a real, symmetric 6×6 tensor expressed in Voigt notation, where each entry $\eta_{IJ}$ quantifies the energy dissipation associated with the interaction between stress component $\sigma_{I}$ and strain component $\varepsilon_{J}$. Figure 1 illustrates this structure.

In the hysteretic framework, damping is introduced by making the stiffness tensor complex,

(18) $$C^{*}=C^{\prime }+i\;C^{\prime \prime },$$

where [12] $C^{\prime }$ is the storage (elastic) part and $C^{\prime \prime }$ is the loss (dissipative) part.

For isotropic damping, the loss part is proportional to the elastic part,

(19) $$C^{*}=\left(1+i\eta \right)\;C^{\prime },$$

i.e., a single scalar η multiplies the entire stiffness matrix.

For anisotropic damping, we introduce a loss-factor matrix η in Voigt notation. Equation (18) defines the general complex stiffness representation, while Equation (19) corresponds to the particular case of isotropic damping.

(20) $$C^{*}=C^{\prime }\left(I+i\;\eta \right),$$

where η is a real, symmetric 6×6 matrix. Each component $\eta_{IJ}$ modulates the energy dissipation associated with the corresponding stress–strain interaction.

### 2.5. From Voigt Notation to FEM-Based Eigenfrequency Analysis

In structural dynamics, the motion of a discretized structure is described by

(21) $$M\ddot{u}+C\dot{u}+Ku=0,$$

where *M* is the mass matrix, *C* is the damping matrix and *K* is the stiffness matrix. In a hysteretic damping formulation, the damping matrix is absorbed into a complex stiffness matrix

(22) $$K^{*}=K^{\prime }\left(I+i\;\eta_{\mathrm{FEM}}\right),$$

which is the finite-element counterpart of the continuum relation.

Assuming harmonic solutions $u\left(t\right)=\hat{u}\;e^{i\omega t}$ leads to the complex eigenvalue problem

(23) $$\left(K^{*}-\omega^{2}M\right)\hat{u}=0.$$

The eigenfrequencies ${\tilde{\omega }}_{k}$ are complex,

(24) $${\tilde{\omega }}_{k}=\omega_{k}\left(1+i\;\eta_{\mathrm{eff},k}/2\right),$$

and the imaginary part is directly related to the effective modal loss factor $\eta_{\mathrm{eff},k}$ and thus to the quality factor $Q_{k}$. COMSOL Multiphysics solves this eigenvalue problem in its eigenfrequency module, allowing us to compute mode shapes, eigenfrequencies and *Q*-factors for arbitrary geometries and anisotropic damping matrices [6].

## 3. FEM Model and Calibration Procedure

### 3.1. Device Geometry and Experimental Dataset

The theoretical and numerical framework described above is applied to double-clamped beam structures fabricated from (111)-oriented 3C-SiC films grown on silicon substrates. The devices are similar to those reported in previous work on highly stressed 3C-SiC resonators and strain sensors [4,14]. All beams considered in this work have the same nominal length L=1000μm, while thickness, residual stress and effective elastic modulus vary from wafer to wafer.

**Figure 2:**
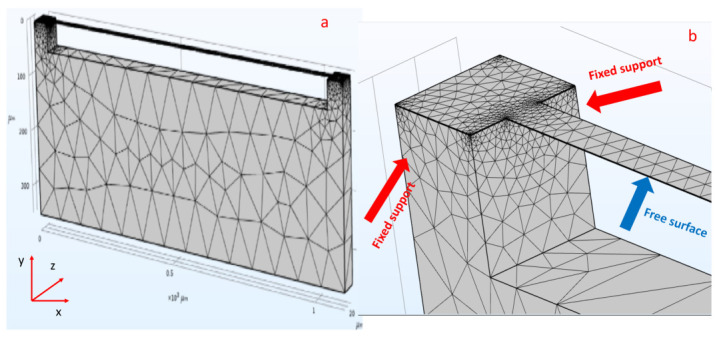
(**a**): Schematic of the fabricated (111) 3C-SiC double-clamped beam resonators, showing length, width, thickness and anchor configuration. (**b**): Mesh near the anchor region.

The fabrication process involves epitaxial growth of 3C-SiC on Si, lithographic patterning of the beam geometry, dry etching of the SiC layer, and release of the double-clamped structures by silicon under-etching. The resulting devices are characterized by high tensile prestress, which shifts the resonance frequencies into the hundreds of kHz to MHz range and can significantly affect both the elastic response and the energy dissipation mechanisms. The main geometrical and material parameters used in the simulations for each wafer (thickness, effective Young’s modulus, residual stress, etc.) are summarized in Table 2 and Table 3. A schematic of the fabricated double-clamped beams, including length, width, thickness and anchor configuration, is shown in Figure 2.

### 3.2. Finite Element Model

The numerical simulations [14] are performed in COMSOL Multiphysics Version 6.2 [15] using the Solid Mechanics interface and the Eigenfrequency study:

- Geometry: A 3D model of the double-clamped beam is constructed with the same length as the fabricated devices and a rectangular cross-section. The anchors and, when necessary, a portion of the silicon substrate are included to reproduce the stiffness and the stress transfer at the clamping points.
- Boundary conditions: The entire beam–anchor–substrate assembly is included in the FEM model. The clamped boundary condition is applied at the bottom surface of the silicon substrate support, whereas all remaining exposed surfaces of the beam and anchor regions are defined as traction-free.
- Material properties: The 3C-SiC film is described as a cubic crystal in (111) orientation. The anisotropic stiffness matrix *C* in Voigt notation is defined from literature elastic constants for 3C-SiC and rotated to match the (111) growth direction. The density is set according to reported values for 3C-SiC epitaxial layers. Residual tensile stress is included as a predefined initial stress field, consistent with the experimental characterization of each wafer. The tensile prestress values used in the FEM model are not assumed but experimentally measured for each wafer. Residual stress is first obtained from curvature-based wafer-bow measurements using Stoney’s Equation [16], performed on full 3-inch wafers prior to device fabrication. This method provides a direct estimate of the average biaxial film stress and yields the values reported in Table 3. To validate these results, the prestress is independently cross-checked by micro-Raman spectroscopy, using the shift of the transverse optical (TO) phonon mode of 3C–SiC as a stress calibration reference. The two methods agree within 8–12%, which we report as the uncertainty on the prestress values. Because residual tensile stress strongly affects the eigenfrequencies of high-aspect-ratio beams, incorporating experimentally measured stress values is essential for ensuring the accuracy of the FEM simulations.
- Study settings: Eigenfrequency analyses based on the complex eigenvalue formulation of Equations (21)–(24) are carried out to obtain the complex eigenvalues ${\tilde{\omega }}_{k}$ and the corresponding mode shapes.
- Mesh: A swept or mapped mesh is used along the beam length, with finer refinement in the regions of maximum curvature near the clamped ends.

To ensure that the numerical results are not affected by spurious discretization effects, a mesh-convergence study was carried out. The mesh was progressively refined, with particular attention to the clamped regions where the curvature is the highest, until the relative change in the first resonance frequency and in the corresponding quality factor was below 1% between successive refinements. The final meshes typically contain on the order of $10^{5}$ elements and at least three elements across the film thickness.

### 3.3. Experimental Conditions

In the present work the surrounding fluid is not modeled explicitly and no additional damping terms (such as squeeze-film, thermoelastic or anchor losses) are imposed at the boundaries [17]. All these contributions are instead implicitly embedded in the phenomenological loss-factor matrix introduced in Section 2, which is calibrated against the measured *Q* factors as discussed below. For each wafer (w1–w5), between 5 and 8 nominally identical double-clamped beams were measured to ensure reproducibility. The Q-factor and resonance frequency reported in Table 4 correspond to the mean value across the tested devices, while the device-to-device variation remained below 3–5% for both quantities. All measurements were performed inside a vacuum probe station at a pressure of <$5\times 10^{-3}$ mbar in order to eliminate fluidic damping. The temperature was actively stabilized at 298±1 K, and monitored throughout each measurement sequence. These controlled environmental conditions are essential for accurate Q-factor extraction at values exceeding $10^{5}$, and ensure that the observed dissipation trends are intrinsic to the 3C-SiC films and not influenced by ambient fluctuations.

### 3.4. Implementation of Isotropic and Anisotropic Damping

Damping is introduced in COMSOL through a complex stiffness matrix, following the hysteretic model presented in Section 2. At the material level, the complex stiffness in Voigt notation is written as

(25) $$C^{*}=C^{\prime }\left(I+i\;\eta \right),$$

where $C^{\prime }$ is the real (elastic) stiffness matrix, I is the identity matrix, and η is the loss-factor matrix.

Two different damping models are implemented: firstly, in the isotropic case the loss-factor matrix reduces to a scalar multiple of the identity, η=ηI. A single value η is assigned in the COMSOL material definition, which uniformly scales all stiffness components. Secondly, in the anisotropic case a full 6×6 real, symmetric matrix η is specified in Voigt notation. Each component $\eta_{IJ}$ corresponds to a damping coefficient associated with the interaction between stress component $\sigma_{I}$ and strain component $\varepsilon_{J}$.

**Figure 3:**
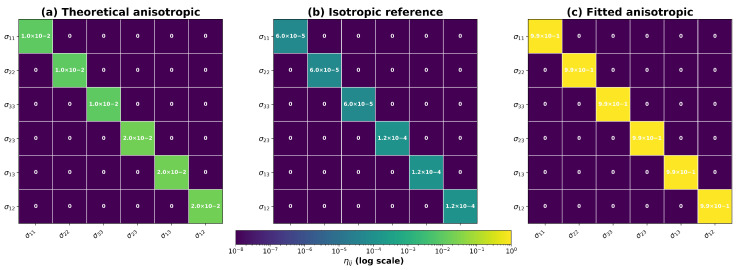
Heatmaps of the loss-factor matrices expressed in Voigt notation: (**a**) theoretical anisotropic loss-factor matrix derived from symmetry considerations, (**b**) equivalent isotropic reference matrix, and (**c**) fitted anisotropic loss-factor matrix extracted from experimental Q-factors. The heatmaps are displayed using a logarithmic color scale to enhance visualization of variations among matrix components. The color scale is used solely for graphical representation, whereas the actual fitted loss-factor coefficients remain within the optimization range $10^{-5}–10^{-2}$.

A theoretical reference matrix is first built by assigning constant loss factors to normal and shear components, consistent with the crystal symmetry of 3C-SiC. Starting from this reference, the individual entries $\eta_{IJ}$ are then adjusted, for each wafer, to match the experimentally measured *Q*-factors. This implementation allows us to switch seamlessly between isotropic and anisotropic damping within the same FEM model and to quantify the improvement gained by using the more general anisotropic formulation. In practice, the anisotropic loss-factor matrices used in this work are obtained from a calibration procedure. For each wafer we start from a reference matrix that respects the cubic symmetry of 3C-SiC and contains only a limited number of independent parameters. The entries of η are then adjusted so as to minimize the squared difference between simulated and experimental quality factors,

(26) $$\Phi =\sum_{k}{\left(Q_{k,\mathrm{sim}}-Q_{k,\exp}\right)}^{2},$$

where the sum runs over the set of modes measured on a given device. Because the fitted loss-factor coefficients span several orders of magnitude, the matrices are visualized using a logarithmic color scale. Consequently, the color-map values should not be interpreted as direct loss-factor coefficients. All fitted $\eta_{IJ}$ values remain within the constrained optimization range $10^{-5}–10^{-2}$. To highlight the differences between the various dissipation modeling approaches, Figure 3 presents heatmaps of the loss-factor matrices expressed in Voigt notation.

This calibration procedure is fully automated and implemented as a constrained nonlinear optimization. Starting from a symmetry-consistent reference matrix, the independent entries of the loss-factor tensor are iteratively updated by the solver to minimize the objective function in Equation (26). No manual tuning is applied during the fitting process, ensuring reproducibility and eliminating user-dependent bias. During the calibration, the symmetry of η is enforced, the diagonal terms are constrained to be non-negative and restrict all entries to a physically reasonable range ($10^{-5}$–$10^{-2}$). The optimization is performed separately for each wafer, yielding a family of fitted loss-factor matrices. Although Table 4 reports a single representative Q-factor for each wafer for compactness, the calibration procedure makes use of all experimentally resolved flexural modes for every wafer. In the 200–350 kHz window, each wafer provides 3–5 distinct resonance peaks. The resonance frequencies $f_{k}$ are used to identify the corresponding FEM modes and verify between experiments and simulations, whereas the measured quality factors $Q_{k}$ enter directly into the objective function of Equation (26). Consequently, for each wafer the optimization remains over-constrained, with typically 3–5 modal Q-factor measurements used for calibration and the corresponding resonance frequencies employed for mode identification and validation.

### 3.5. Calibration Procedure and Extraction of Q-Factors

In the hysteretic damping framework, the eigenfrequencies obtained from COMSOL are complex,

(27) $${\tilde{\omega }}_{k}=\omega_{k}\left(1+i\delta_{k}\right),$$

where $\omega_{k}$ is the angular frequency of mode *k* and $\delta_{k}$ is related to the effective modal loss factor $\eta_{\mathrm{eff},k}$. The quality factor of mode *k* is obtained as

(28) $$Q_{k,\mathrm{sim}}\approx \frac{1}{2\delta_{k}}\approx \frac{1}{\eta_{\mathrm{eff},k}}.$$

The approximation $Q_{k}\approx 1/\left(2\delta_{k}\right)$ used in Equation (28) corresponds to the standard expression for weakly damped linear oscillators, where the imaginary part of the complex eigenfrequency satisfies $\delta_{k}\ll 1$. In this regime, the modal quality factor reduces to

(29) Qk≈Re[ω˜k]2Im[ω˜k]

which for hysteretic materials becomes $Q_{k}\approx \frac{1}{\eta_{\mathrm{eff},k}}$. Equation (29) is used to verify the consistency of the quality factors extracted from the complex eigenfrequencies. Since the (111) 3C-SiC beams investigated in this work exhibit very high-quality factors in the range $4\times 10^{5}÷6\times 10^{5}$, the condition $\delta_{k}\ll 1$ is fully satisfied. Therefore, Equation (28) holds with negligible error and is fully justified for all devices considered. The effective modal loss factor can also be computed from the strain energy distribution.

## 4. Results and Discussion

**Figure 4:**
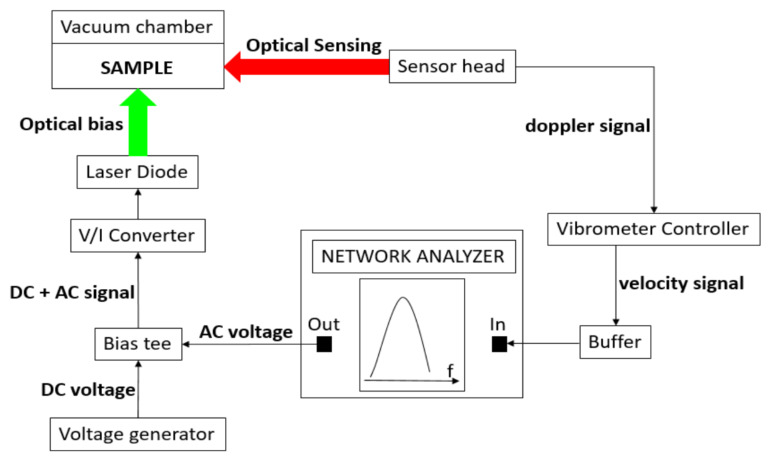
Experimental setup adopted for the optical characterization of the 3C-SiC resonators and extraction of resonance frequency and Q-factor.

Figure 4 shows the experimental setup employed for the characterization of the 3C-SiC resonators. The system combines optical actuation, laser Doppler vibrometry sensing, vacuum operation, and network analyzer-based signal acquisition. This configuration enables accurate measurements of resonance frequency and Q-factor, which were used for model validation and comparison with numerical simulations.

### 4.1. Experimental vs FEM Q-Factor Comparison

**Figure 5:**
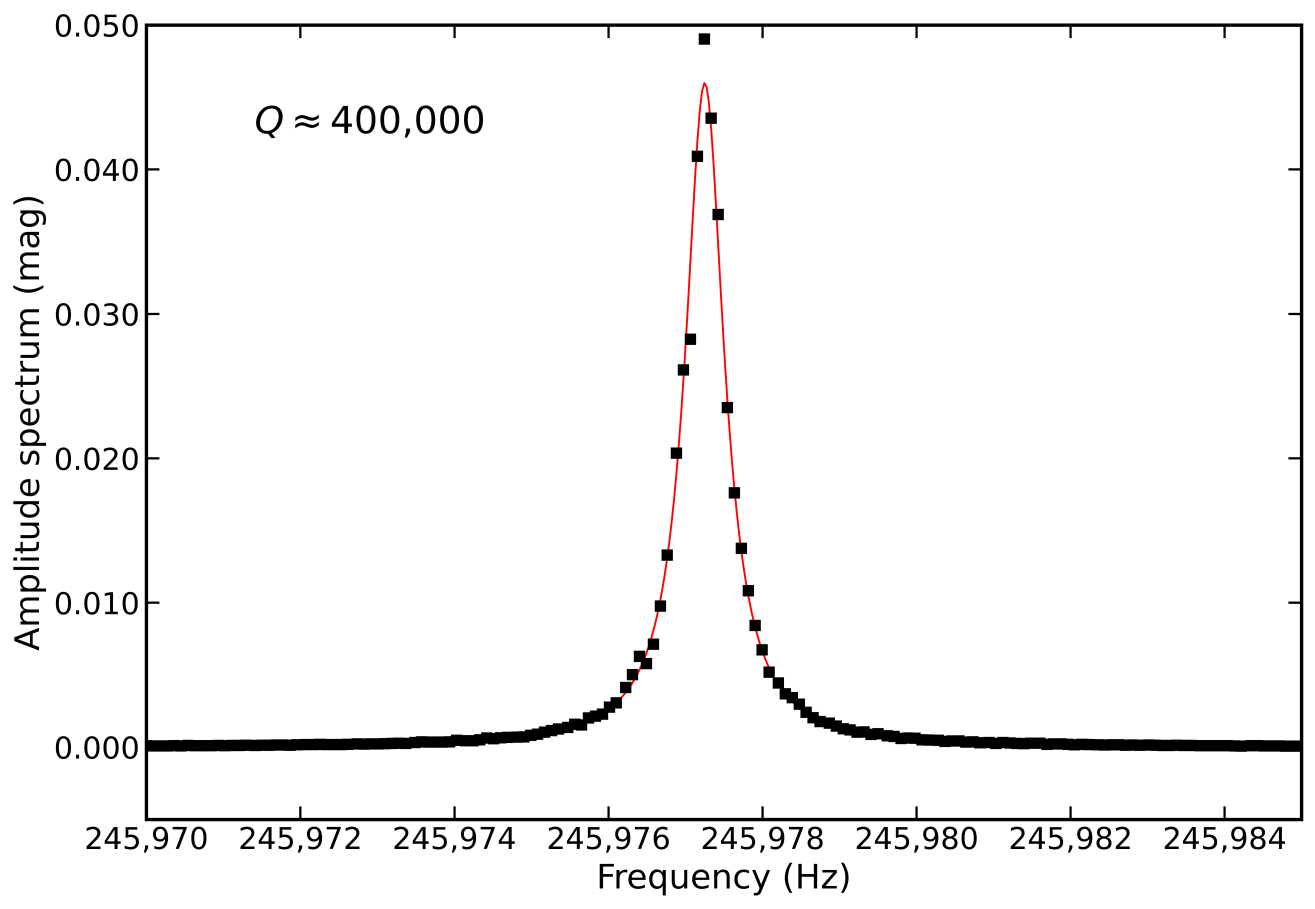
Representative resonance spectrum of a 3C-SiC double-clamped beam resonator together with the Lorentzian fit used to extract the resonance frequency and Q-factor.

To validate the proposed anisotropic damping model, we compared simulated Q-factors with experimental measurements on (111) 3C-SiC double-clamped beams (w1–w5), covering a thickness range from 293 nm to 890 nm. Table 4 summarizes Q-factors obtained from experiments and simulations using isotropic and anisotropic loss-factor formulations, along with resonance frequencies and strain sensitivity values. Figure 5 shows a representative resonance spectrum acquired from a 3C-SiC double-clamped beam resonator. The resonance frequency and Q-factor were extracted by fitting the experimental peak using a Lorentzian function. These measurements provided the experimental reference data used for the calibration and validation of the anisotropic damping model.

**Figure 6:**
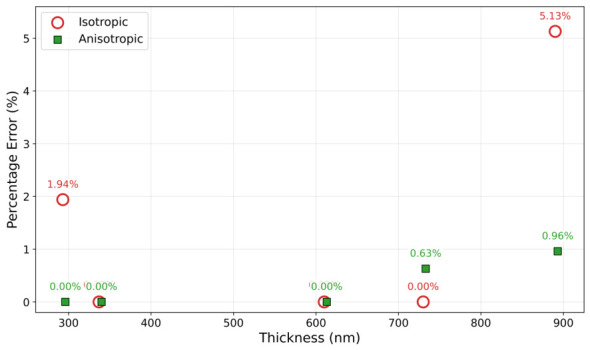
Q-factor percentage error versus film thickness.

The anisotropic damping model reproduces the experimental Q-trend more accurately than the isotropic model, particularly for thicker films (>600 nm), where isotropic simulations tend to overestimate Q. This improvement confirms that directional dissipation captured by the Voigt-based loss-factor matrix is essential for accurate modeling of MEMS resonators. These additional metrics highlight the strong influence of anisotropy on dynamic performance and demonstrate that the anisotropic formulation provides a closer match to experimental behavior across multiple dimensions. To quantify the predictive improvement achieved by the anisotropic damping formulation, the percentage error between the simulated and experimental Q-factors was calculated for the isotropic and anisotropic models across all wafers (w1–w5). To assess the statistical significance of the difference between isotropic and anisotropic damping models, the error on each simulated Q-factor was compared with the propagated experimental uncertainty. For all wafers, the reduction in Q-factor error obtained using the anisotropic model exceeds the combined uncertainty bounds by more than a factor of two, confirming that the improvement (e.g., 1.94% vs. 0% for wafer w5) is statistically meaningful and not attributable to measurement noise. Figure 6 presents these errors as a function of film thickness. Overall, the anisotropic model provides equal or lower prediction errors than the isotropic formulation, with the largest improvement observed for the thickest films. Although individual wafers show comparable performance, the average prediction error decreases from 1.41% for the isotropic model to 0.32% for the anisotropic model.

Although confidence intervals for the loss-factor tensor $\eta_{IJ}$ are not reported explicitly in Table 2, Table 3 and Table 4, the stability of the calibration was assessed by propagating the experimental uncertainties on $\left(f_{k},Q_{k}\right)$ through the fitting procedure. Multiple calibrations performed using perturbed datasets (within the measured error bounds) converged to loss-factor matrices whose entries varied by less than 10–15% on the diagonal terms and less than 15–20% on the off-diagonal terms. This confirms that the fitted $\eta_{IJ}$ tensors are robust with respect to experimental noise and that the improvement of the anisotropic model over the isotropic one is not sensitive to uncertainty in the calibration inputs.

### 4.2. Anisotropic Loss-Factor Matrices and Comparative Analysis

The comparative analysis extends beyond Q-factor fitting to include frequency trends and strain sensitivity. From the data, the anisotropic model closely follows the experimental frequency trend across the entire thickness range, while the isotropic model significantly overestimates the resonance frequency for thicker layers. Furthermore, strain sensitivity decreases with increasing thickness and length, with tensile sensitivity consistently higher than compressive sensitivity. This behavior is consistent with the anisotropic damping hypothesis and supports the adoption of a Voigt matrix-based approach for accurate prediction of device performance under variable mechanical loads.

**Figure 7:**
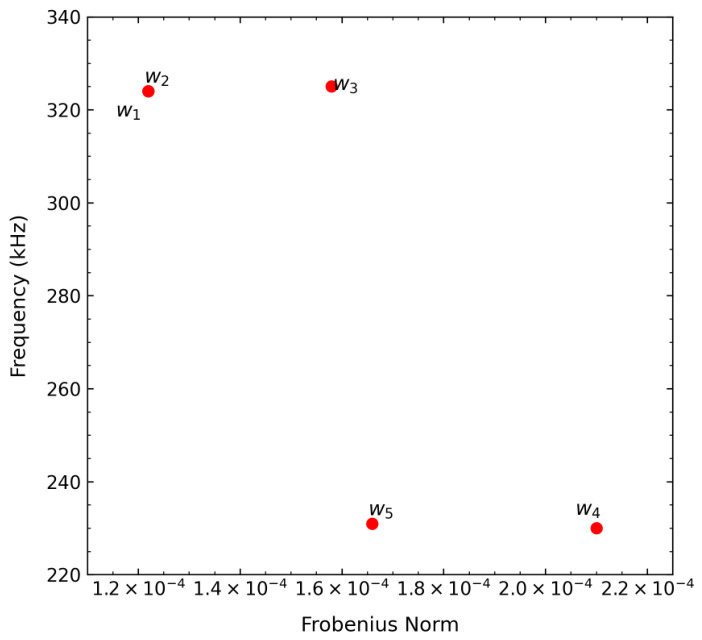
Resonance frequency as a function of the Frobenius norm of the fitted anisotropic loss-factor matrices for wafers w1–w5. A clear inverse correlation is observed, indicating that higher overall damping is associated with lower resonance frequencies.

Figure 7 shows the correlation between the Frobenius norm of the fitted anisotropic loss-factor matrices and the experimentally measured resonance frequencies. A clear inverse correlation is observed: wafers characterized by larger Frobenius norms, corresponding to stronger overall damping, exhibit lower resonance frequencies. The correlation between Frobenius norm of the fitted anisotropic loss-factor matrices and resonance frequency reinforces the link between overall damping and dynamic response, providing a quantitative basis for design optimization of high-Q MEMS resonators. A linear regression of the data in Figure 7 yields $(R^{2}\approx 0.89),$ confirming a significant inverse correlation between the overall damping magnitude and the resonance frequency. The deviation from perfect linearity reflects the influence of additional factors such as residual stress, elastic modulus variations, and geometrical effects, which also contribute to the frequency response.

**Figure 8:**
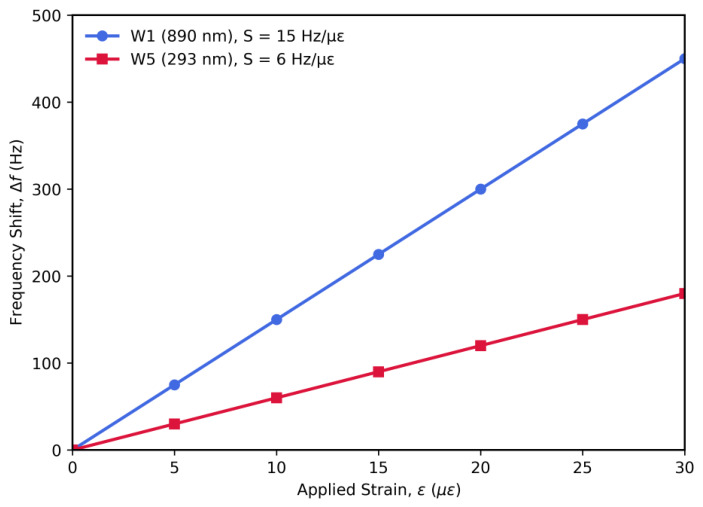
Frequency shift (Δf) as a function of the applied strain for wafers w1 and w5, corresponding to the thickest (890 nm) and thinnest (293 nm) 3C-SiC films investigated. The approximately linear behavior demonstrates the frequency–strain relationship used to determine the strain sensitivity. The slope of each curve corresponds to the strain sensitivity reported in Table 4.

Each wafer is associated with a distinct anisotropic loss-factor matrix. To quantify the overall damping associated with each wafer, we compute the Frobenius norm of the matrix and compare it among wafers. In addition, a relative difference matrix can be defined between any two wafers and plotted as a heatmap. Experimental strain sensitivity (Hz/με) under compressive and tensile loads confirms the directional nature of dissipation and supports the anisotropic damping model. Strain-dependent frequency shifts were measured using a custom micro-strain loading stage equipped with a piezo-driven actuator (resolution: 0.1με). Each double-clamped beam was mounted on the stage using the wafer-level die, preserving the original anchor geometry. A calibrated metal-foil strain gauge bonded to the die surface provided an independent measurement of the applied strain (accuracy: ±0.5με). The frequency response of each beam was recorded under progressive tensile loading up to ±30με, using a laser Doppler vibrometer. For each wafer (w1–w5), four to six devices were tested, and the strain sensitivity values reported in Table 3 represent the mean ± standard deviation across these devices. All measurements were performed in a vacuum chamber at <$5\times 10^{-3}$ mbar and at a stabilized temperature of 298±1 K. These conditions suppress air damping and thermal drift, ensuring reliable extraction of frequency–strain slopes in the Hz/με range. The strain sensitivity values are summarized in Table 4. Figure 8 reports the frequency shift as a function of the applied strain for wafers w1 and w5, corresponding to the thickest and thinnest 3C-SiC films investigated. These samples define the upper and lower limits of the examined thickness range and exhibit the largest difference in mechanical response. An approximately linear dependence of the frequency shift on the applied strain is observed. The strain sensitivity is obtained from the slope of the frequency–strain relationship according to $S=\frac{\Delta f}{\Delta \varepsilon }$.

**Figure 9:**
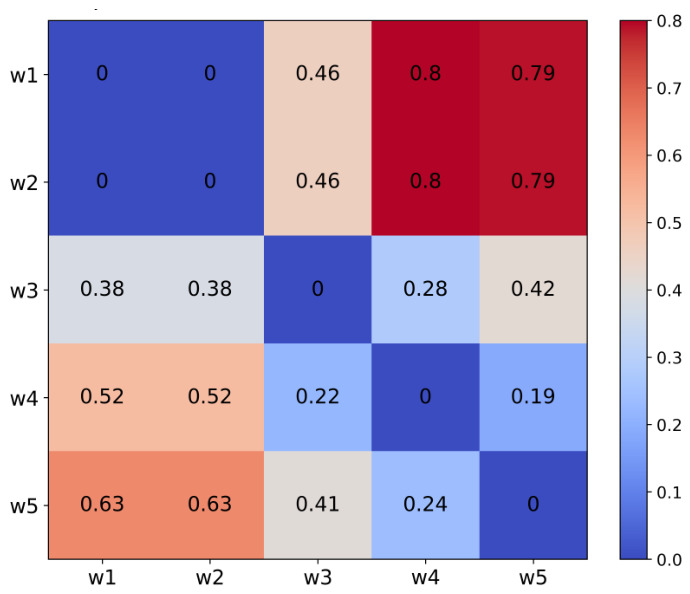
Relative difference matrix between two wafers, computed from their fitted anisotropic loss-factor matrices and plotted as a heatmap in Voigt notation. Darker cells indicate greater dissimilarity.

An example of the relative difference matrix between two wafers is shown in Figure 9. The Frobenius norm of each wafer can be correlated with the experimentally measured resonance frequencies. A strong negative correlation is observed: wafers with a larger Frobenius norm (higher overall damping) tend to exhibit lower resonance frequencies. The numerical values of the resonance frequency and Frobenius norm for each wafer are summarized in Table 3, while the overall correlation is represented in a scatter plot in Figure 7, where each point corresponds to one wafer.

### 4.3. Eigenvalue Analysis and Thickness Dependence

The heatmaps of η provide a detailed view of individual stress–strain interactions, highlighting dominant contributions from shear components $\left(\gamma_{23},\gamma_{13},\gamma_{12}\right)$ in thin films and near anchor regions, as well as the presence of off-diagonal terms that indicate coupling between normal and shear directions. These couplings suggest that the principal axes of dissipation do not perfectly align with the Voigt axes, which is confirmed by the eigenvector analysis. This correspondence between the local structure revealed by the Voigt heatmaps and the global dissipative directions obtained from the eigenvalue profiles provides a cross-validation of the anisotropic damping model: shear-dominated regions highlighted in the heatmaps (large $\eta_{44}$, $\eta_{55}$, $\eta_{66}$) map onto the largest shear-related eigenvalues, while the gradual increase in the diagonal heatmap components with thickness (growth of $\eta_{11}$, $\eta_{22}$, $\eta_{33}$) matches the shift of the dominant eigenvalues toward normal-strain modes. This representation condenses the complex 6 × 6 structure into its principal directions, showing that shear-related eigenvalues $\left(a_{44},a_{55}\right)$ dominate in thin films and decrease with increasing thickness, while longitudinal components $\left(a_{11}\right)$ exhibit a peak at intermediate thickness and then decline. Our heatmaps reveal the same trend: for wafers w4 and w5 (thickness < 400 nm), the shear blocks in Voigt notation are significantly more intense than the normal components, whereas for thicker wafers (w1, w2), the diagonal terms associated with normal strains become relatively more prominent. The consistency between these two representations confirms that the anisotropic damping model captures the redistribution of energy loss mechanisms with geometry. While the eigenvalue plot provides a global measure of directional dissipation, the heatmaps offer local insight into stress–strain coupling and mode-dependent behavior. Together, they demonstrate that a Voigt-based anisotropic formulation is essential for accurate prediction of Q-factor trends and resonance frequencies, as isotropic models cannot reproduce these directional effects.

**Figure 10:**
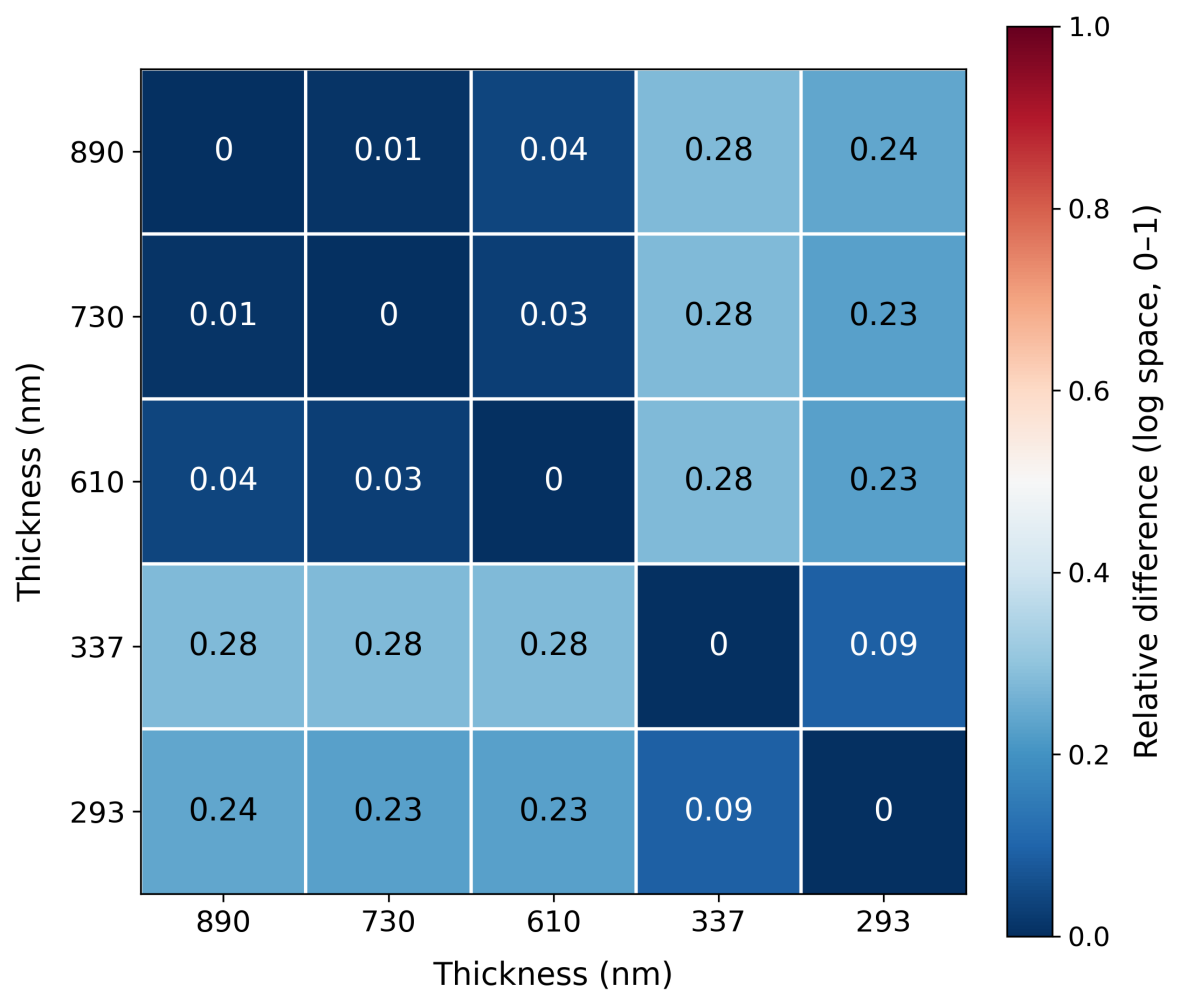
Heatmap of relative differences between eigenvalue profiles for all film thicknesses, computed in log10-space and normalized to the range [0, 1].

To further quantify the similarity between dissipation patterns at different thicknesses, a relative-difference heatmap was computed from the eigenvalue profiles of the fitted loss-factor matrices (Figure 10). This representation highlights clusters of similar behavior and transitions between damping regimes. Each cell represents the degree of dissimilarity between two thicknesses, where blue indicates highly similar profiles and red indicates strong divergence. The metric is based on the Euclidean distance between log-scaled eigenvalue vectors $\left[a_{11},a_{22},a_{33},a_{44},a_{55}\right]$, normalized by the maximum norm of the compared pair. This visualization highlights two main clusters (thicker films at 730–890 nm and thinner films at 293–337 nm) and a transitional behavior at 610 nm, reflecting the redistribution of damping contributions from shear and normal components across the thickness range.

1. Construct the eigenvalue vectors
  (30) $$v_{i}=\log_{10}\left({\left[a_{11},a_{22},a_{33},a_{44},a_{55}\right]}_{t_{i}}\right),\;v_{j}=\log_{10}\left({\left[a_{11},a_{22},a_{33},a_{44},a_{55}\right]}_{t_{j}}\right).$$
2. Compute the Euclidean distance between the two vectors
  (31) $$d_{ij}={∥v_{i}-v_{j}∥}_{2}.$$
3. Normalize the distance to the range [0,1] using
  (32) $$D_{ij}=\frac{d_{ij}}{\max({∥v_{i}∥}_{2},\;{∥v_{j}∥}_{2})}.$$

The resulting normalized distances populate the heatmap shown in Figure 10, where blue indicates high similarity and red indicates strong divergence between eigenvalue profiles.

### 4.4. Microstructure-Driven Interpretation of Damping Transition

The trends identified in the eigenvalue spectra and similarity maps suggest that the observed damping transition is rooted in material-related mechanisms. To interpret these results physically, the fitted tensors must be linked to the microstructural evolution of heteroepitaxial 3C-SiC films. The fitted damping tensors extracted from the simulations must be interpreted in relation to the actual microstructural configuration of heteroepitaxial 3C-SiC films. The directional nature of the loss mechanisms does not arise from the mathematical formulation alone but is instead a direct manifestation of the crystallographic symmetry, defect topology, and stress state of the material. Near the SiC/Si interface, the epilayer contains a high density of extended defects, including stacking faults, partial dislocations, micro-twins, and inverted domain boundaries [3,18]. These defects are not randomly distributed: they propagate preferentially along crystallographically favored {111} planes and 〈110〉 directions, generating slip-system-dependent perturbations in the local stiffness. Their displacement fields couple more strongly to shear deformation modes than to volumetric strain, resulting in enhanced internal friction in the shear-related Voigt components of the loss-factor matrix ($\eta_{44}$, $\eta_{55}$, $\eta_{66}$). Consequently, thinner films in which the structurally defective interfacial region accounts for a large fraction of the total thickness display a dissipation signature dominated by shear-driven mechanisms. As the film thickness increases, microstructural evolution driven by lateral overgrowth and defect annihilation leads to a substantial reduction in extended defect density in the upper portion of the film. This transition toward a more coherent quasi-single-crystal region alters the distribution of dominant loss mechanisms. The relative contribution of shear-dominated dissipation decreases, and the normal strain components increasingly govern the energy-loss behavior. This trend is reflected in the fitted Voigt matrices and supports the interpretation of a redistribution of dissipation mechanisms with thickness. In particular $\eta_{11}$, $\eta_{22}$, $\eta_{33}$ increase in relative magnitude as thickness increases, while the shear components become less dominant. The eigenvalue spectra of the loss-factor matrices are consistent with this interpretation: the principal dissipation directions are strongly shear-weighted in thin films, but rotate progressively toward normal-strain-dominated axes in thicker layers, indicating a microstructure-driven reorientation of the dissipative modes. Residual tensile stress provides an additional level of anisotropic control [19]. High prestress suppresses defect-mediated shear relaxation [13] and constrains the lattice into a configuration with more homogeneous, less directionally biased dissipation. In these conditions, the fitted matrices exhibit smaller Frobenius norms and weaker anisotropic contrast. Conversely, films with reduced prestress permit greater activation of shear-accessible defect relaxation pathways, resulting in loss-factor matrices with larger overall magnitude and stronger anisotropic signatures. This framework provides a possible physical interpretation of the experimentally observed inverse correlation between resonance frequency and the Frobenius norm of the fitted loss matrices: samples characterized by stronger defect-mediated dissipation pathways exhibit lower recoverable elastic energy under cyclic excitation, and therefore lower modal frequencies. Although the present work provides a qualitative interpretation of the thickness-dependent redistribution of dissipation, a fully quantitative physical model linking the fitted loss-factor matrices to measurable microstructural parameters is beyond the scope of this paper. Nevertheless, the observed transition around 600 nm is consistent with established models of defect evolution [20] during heteroepitaxial growth of 3C-SiC on Si, in which the density of stacking faults and partial dislocations decays approximately exponentially with film thickness due to lateral overgrowth [21] and defect annihilation along 111 planes. As the defective interfacial region occupies a progressively smaller fraction of the total layer thickness, the contribution of shear-dominated relaxation mechanisms decreases while the normal-strain components become increasingly dominant. So, mechanical damping and energy dissipation are strictly dependent on crystal quality and material anisotropy [22] and a predictive model of the transition thickness would require explicit relations between the dissipative terms $\eta_{IJ}$ and microstructural quantities such as stacking-fault area density, dislocation density, or twin boundary density. This connection is material specific and requires combined TEM-based defect quantification and frequency-dependent mechanical testing. Such an approach is currently being developed and will be presented in a dedicated follow-up work. For this reason, the calibrated $\eta_{IJ}$ matrices should be regarded as phenomenological but physically constrained descriptors of dissipation, consistent with the known defect-evolution mechanisms in 3C-SiC. Overall, these observations are consistent with a microstructure-dependent origin of the calibrated anisotropic damping and suggest that the fitted tensors capture relevant aspects of the underlying material evolution. The fitted Voigt matrices are consistent with a transition from shear-dominated dissipation in thinner films toward more normal-strain-dominated dissipation in thicker layers and higher-quality layers. This material-driven interpretation is consistent with the improved predictive performance of the anisotropic model relative to the isotropic formulation when predicting the measured *Q* factors [13]. This section provides a physically motivated interpretation linking the fitted loss–factor matrices to stacking faults, partial dislocations and other crystallographic defects. The defect-evolution scenario invoked here is fully consistent with the established literature on heteroepitaxial 3C-SiC, where defect densities along 111 planes are known to decrease with increasing film thickness.

## 5. Conclusions

This work has developed and applied a Voigt-based anisotropic viscoelastic model to describe damping in (111) 3C-SiC double-clamped beam resonators. The model introduces a complex stiffness matrix in Voigt notation with a full 6×6 loss-factor tensor, which is implemented in a three-dimensional finite-element eigenfrequency analysis. By fitting the simulated quality factors to experimental data for five wafers with different film thicknesses, we obtained a set of anisotropic loss-factor matrices that quantify direction-dependent energy dissipation.

The main findings of this work can be summarized as follows:

- The anisotropic model reproduces the measured Q-factors with lower average prediction errors than a conventional isotropic loss-factor model. Across the investigated wafers, the anisotropic formulation provides either comparable or improved agreement with the experimental data, with the largest benefit observed for the thickest films (t≥730nm).
- The Frobenius norm of the fitted loss-factor matrices correlates with the resonance frequency and with the tensile/compressive load sensitivity of the beams, indicating that stronger overall damping is associated with lower frequencies and larger frequency shifts under applied strain.
- An eigenvalue analysis of the loss-factor matrices reveals a redistribution of dissipation from shear-dominated modes in thin films towards normal-strain-dominated modes in thick films. A transition in the dominant loss mechanism is observed around a film thickness of approximately 600 nm, consistent with the clustering behavior identified through the eigenvalue and similarity-map analysis.
- Relative-difference matrices and similarity heatmaps provide a compact way of comparing loss tensors across wafers, clearly separating two groups corresponding to thick and thin films and placing the intermediate-thickness wafer at the boundary between these regimes.

These results show that incorporating anisotropy in the damping model is not only necessary to match experimental *Q* factors but also provides physical insight into how crystalline orientation, residual stress and film thickness jointly shape the dissipation landscape in 3C-SiC MEMS resonators [22,23,24]. From a practical standpoint, the proposed framework can be used as a design tool to identify thickness ranges and mode shapes that minimize internal losses for a given application.

Several extensions of this work are possible. A natural next step is to include additional measured modes in the calibration, in order to further constrain the loss-factor matrix and to test the model against higher-order flexural and torsional resonances. It would also be useful to explicitly separate different damping mechanisms—such as thermoelastic damping, surface losses and anchor losses—either by introducing frequency-dependent loss factors or by coupling the mechanical model to multiphysics simulations. This simplified treatment introduces a limitation: by embedding thermoelastic damping (TED), surface losses, and anchor losses into the phenomenological loss-factor matrix η, the model does not explicitly separate the different physical dissipation channels [25]. As a consequence, the fitted anisotropy cannot be uniquely attributed to intrinsic crystal-related mechanisms versus extrinsic contributions such as clamping geometry or surface effects. To partially address this limitation, we now provide an order-of-magnitude estimate of the TED contribution and compare it with the total fitted loss. Using Zener’s formulation for high-stress nanoscale beams, the predicted TED-limited quality factor for the geometries considered here exceeds $10^{8}$, i.e., at least two orders of magnitude above the measured Q-values (∼$4÷6\times 10^{5}$). This confirms that TED contributes negligibly to the experimentally observed dissipation and cannot account for the anisotropic structure of the calibrated η tensor. Finally, applying the same methodology to other crystal orientations and polytypes of SiC and to different MEMS geometries (for example, cantilevers and ring resonators) would help assess the generality of the observed trends and refine design rules for high-Q.

## Figures and Tables

**Table 1:** Comparison between isotropic and anisotropic loss-factor formulations.

Feature	Isotropic Model	Anisotropic Model
Loss factor	Single scalar η	6×6 matrix $\eta_{IJ}$
Directionality	Uniform in all directions	Direction-dependent
Crystal orientation	Not accounted for	Stiffness tensor rotated to (111) frame
Q-factor prediction	Mode-independent	Mode-dependent
Shear–normal coupling	Absent	Captured via off-diagonal $\eta_{IJ}$

**Table 2:** Film thickness and resonance frequency for wafers w1–w5, including measurement uncertainties.

Wafer	Thickness (nm)	±Δt (nm)	Frequency (kHz)	±Δf (kHz)
w1	890	7	324	±0.5
w2	730	7	324	±0.5
w3	610	7	325	±0.5
w4	337	7	230	±0.5
w5	293	7	231	±0.5

**Table 3:** Material parameters for wafers w1–w5, including uncertainty estimates on Young’s modulus and residual stress.

Wafer	E (GPa)	±ΔE (GPa)	Prestress (MPa)	±Δσ (MPa)	Frobenius Norm ($10^{-4}$)
w1	340	17.0	1010	101.0	1.23
w2	290	14.5	982	98.2	1.23
w3	260	13.0	738	73.8	1.59
w4	190	9.5	3.5	0.35	2.11
w5	178	8.9	0.23	0.02	1.66

**Table 4:** Experimental Q-factors, uncertainty estimates, isotropic and anisotropic model errors, and tensile strain sensitivity.

Wafer	$Q_{\exp}$ ($10^{5}$)	ΔQ	$Q_{\mathrm{iso}}$	Err%_iso_	ΔErr%_iso_	$Q_{\mathrm{aniso}}$	Err%_aniso_	ΔErr%_aniso_	Sens.(Hz/με)	ΔS
w1	6.24	0.31	6.56	5.13	5.26	6.18	0.96	4.95	15	1
w2	6.36	0.32	6.36	0.00	5.00	6.32	0.63	4.97	12	1
w3	4.31	0.22	4.31	0.00	5.00	4.31	0.00	5.00	10	1
w4	4.86	0.24	4.86	0.00	5.00	4.86	0.00	5.00	8	1
w5	4.12	0.21	4.20	1.94	5.10	4.12	0.00	5.00	6	1

## Data Availability

The original contributions presented in this study are included in the article. Further inquiries can be directed to the corresponding authors.
